# S-FAME: a low-threshold municipal social services intervention for digital media–related family difficulties in children aged 10–14 years: protocol for a randomized controlled trial

**DOI:** 10.1186/s40359-026-05302-x

**Published:** 2026-08-12

**Authors:** Sabina Kapetanovic, Emma Claesdotter-Knutsson

**Affiliations:** 1 Department of Behavioral Studies, University West, Trollhättan, Sweden; 2 Department of Psychology, University of Inland, Lillehammer, Norway; 3 Child and Adolescent Psychiatry, Psychiatry, Department of Clinical Sciences Lund, Lund University, Lund, Sweden; 4 Child and Adolescent Psychiatry, Skane University Hospital, Lovisastigen 11, Lund, 22241 Sweden

**Keywords:** Digital media, Early intervention, Family support, Health economics, Implementation, Parent-child relationship, Problematic gaming, Randomized controlled trial, Screen-related family conflict, Social services

## Abstract

**Background:**

Problematic gaming and broader digital media-related family difficulties are increasingly encountered not only in specialist mental health services but also in everyday municipal support services. Sweden’s new Social Services Act strengthens municipalities’ responsibility to provide early, accessible, and knowledge-based support, creating both an opportunity and an obligation to evaluate whether structured, family-centered programs can be delivered effectively in this setting. The present protocol therefore addresses whether S-FAME, a social-services adaptation of the FAME model, can be implemented and evaluated as a low-threshold, preventive support within Swedish municipal social services. This randomized controlled trial aims to evaluate the effectiveness, implementation feasibility, acceptability, and health economic consequences of S-FAME. Specifically, the trial will test whether S-FAME reduces child screen-related problems and improves parent-child relationship outcomes compared with treatment as usual followed by access to a brief parent-focused digital intervention, and will examine whether changes in parenting behaviors mediate changes in child outcomes.

**Methods:**

Families with children aged 10–14 years who seek municipal social services support because of digital media use (including gaming and excessive social media use) or related family conflict will be recruited from participating municipalities. Atleast176 families will be randomized in a 1:1 ratio to immediate S-FAME or a condition consisting of regular support from social services during the study period and subsequent access to P-FAME, a three-session online intervention for parents. S-FAME is delivered by trained municipal staff and retains selected family-centered principles from FAME while adapting content, referral pathways, risk management, and implementation procedures to preventive social services. Quantitative outcomes will be collected at baseline, postintervention, and 1-month follow-up. Qualitative interviews and focus groups will explore family and staff experiences, implementation fit, and the balance between fidelity and local adaptation. A health economic evaluation will estimate implementation costs and potential cost-effectiveness from municipal and broader societal perspectives.

**Discussion:**

Recruitment is planned to begin in February 2026 and end in December 2026. Final analyses of trial outcomes, qualitative implementation data, and health economic data are planned for the first quarter of 2027.

**Trial registration:**

ClinicalTrials.gov NCT07245862 approval date April 20, 2026 (ongoing trial).

**Supplementary Information:**

The online version contains supplementary material available at 10.1186/s40359-026-05302-x.

## Introduction

Children’s digital media environments have become embedded in everyday development rather than representing a discrete or exceptional exposure. Gaming, social networking, streaming, messaging, and school-related digital activities now intersect with peer relationships, leisure, learning, sleep routines, and family life. A scientific rationale for intervention therefore requires a distinction between high-frequency use and clinically or socially problematic use. In ICD-11, gaming disorder is defined by impaired control over gaming, increasing priority given to gaming over other activities, and continuation or escalation despite negative consequences, with sufficient severity to impair personal, family, social, educational, occupational, or other important functioning, usually over at least 12 months [1]. In community and preventive settings, however, families often seek support before a child meets diagnostic criteria—presenting instead with recurrent conflict, disrupted routines, sleep problems, emotional distress, reduced participation in offline activities, or impaired school and family functioning [2, 3].

Recent population and public health data indicate that problematic digital media use is relevant beyond specialist psychiatric services. In the WHO Health Behaviour in School-aged Children (HBSC) study across Europe, central Asia, and Canada [4], problematic social media use among adolescents increased from 7% in 2018 to 11% in 2022, and 12% of adolescents were classified as being at risk of problematic or risky gaming; boys were more likely than girls to be at risk of problematic gaming, whereas girls reported higher rates of problematic social media use [4]. Swedish reports similarly show that digital media are deeply integrated into child and adolescent lives, with widespread use of social media and gaming among young people [5, 6]. These patterns do not imply that ordinary digital media use is inherently harmful. Rather, they underscore the need to identify the subgroup of families for whom digital media routines have become difficult to regulate and are associated with distress, impairment, or escalating parent-child conflict.

The Swedish Public Health Agency has emphasized that children’s digital media use should be understood in relation to sleep, physical activity, schoolwork, social relationships, and family routines rather than as screen time alone [2, 3]. This orientation is consistent with developmental and family-systems perspectives. According to transactional models of development [7, 8], child behavior and the family environment influence each other over time, with each shaping and being shaped by the other across developmental contexts. In the context of digital media use, parental monitoring, boundary setting, emotional warmth, family communication, and conflict may shape children’s media habits, while persistent screen-related problems may also increase parental stress and undermine relationship quality. Systematic reviews support this bidirectional and contextual interpretation: poorer parent-child relationship quality, negative family dynamics, and lower parental involvement have been associated with problematic gaming and problematic internet use, whereas positive parenting and supportive family dynamics appear protective [9, 10]. Longitudinal work further suggests that family dysfunction and problematic gaming may reinforce one another across time [11]. These bidirectional dynamics are particularly relevant for sleep, school functioning, and peer relationships, which may serve as both antecedents and consequences of problematic digital media use within the family system [11].

Although preventive programs targeting problematic digital media use are on the rise, they generally focus on the child alone, without considering the broader social contexts in which the child and their behaviors are embedded. A family-centered intervention is therefore theoretically justified: the target is not only the child’s digital behavior but also the routines, communication patterns, and parenting practices through which digital media use is negotiated. Our previous clinical studies on problematic gaming have shown that both children and parents describe the parent-child relationship as central when gaming becomes problematic, and that families request support that includes parents rather than focusing exclusively on the child [12–17].

The original FAME program was developed for CAMHS (Child and adolescent psychiatry health services) as a group-based, family-centered intervention for children and adolescents with problematic gaming or excessive screen use and their parents aim at. It was informed by theoretical and empirical knowledge of parenting and child and adolescent development, findings from an earlier randomized trial of individual treatment for youth gaming problems, and interviews with parents about their support needs. FAME combines psychoeducation, cognitive restructuring, social-emotional learning, and parenting skills through an individual preparatory meeting followed by four group sessions for children and parents. The sessions address the positive and negative aspects of screen use and boundary setting, links between thoughts, feelings, and behaviors, conflict triggers and emotion regulation, and sleep hygiene [18].

However, the pathway through specialist child and adolescent psychiatry may be too narrow for families who experience screen-related conflict but do not require, or are not ready to seek, psychiatric treatment. This creates a prevention and service gap between general public health advice and specialist clinical intervention [18]. Together, these findings underscore the need for an intermediate, family-centered support pathway that is accessible, nonstigmatizing, and grounded in evidence. Although preventive programs targeting digital media use are emerging [10, 14], they generally focus on the child alone and do not address the broader family context. The present study is situated in that gap—between universal public health messaging and specialist psychiatric care—by evaluating S-FAME (where S stands for Social services) as a structured, family-centered program delivered within Swedish municipal social services.

To address this gap, we developed S-FAME, a family-centered program adapted for preventive social services. S-FAME is designed as a low-threshold municipal social services intervention for families with children aged 10–14 years who experience problematic gaming, excessive screen use, or broader digital media-related family difficulties. This setting is scientifically and practically important. Sweden’s new Social Services Act, effective from July 2025, emphasizes preventive, accessible, and knowledge-based social services, and gives municipalities greater scope to provide some interventions without an individual needs assessment [19, 20]. Swedish municipal social services already deliver a range of evidence-based parenting support programs, making them a natural setting for a family-centered digital media intervention. Evaluating S-FAME in this context will therefore address not only whether a family-centered model can improve child and family outcomes, but also whether such support can be implemented as an early, nonstigmatizing, and scalable municipal service.

## Objectives

The overarching aim is to evaluate S-FAME as an adapted, municipal social services intervention for families with children aged 10–14 years experiencing digital media-related conflict, problematic gaming, or excessive screen use.

The specific research questions are as follows:

1. Does S-FAME reduce child digital media-related problems compared with the comparator pathway?
2. Does S-FAME improve parenting practices, parent–child communication, and perceived warmth and conflict in the family?
3. Are changes in parenting behavior and family interaction associated with changes in child screen-related outcomes?
4. Is S-FAME feasible, acceptable, and appropriate for delivery by municipal social services staff?
5. What implementation conditions support or hinder delivery, fidelity, family engagement, and scale-up?
6. What are the costs and potential cost-effectiveness of S-FAME from municipal and societal perspectives?
7. What are the experiences of children and families who participate in S-FAME, and how do they understand and engage with the support offered through social services?

### Methods

This protocol was prepared in accordance with the Standard Protocol Items: Recommendations for Interventional Trials (SPIRIT) 2013 statement [21]. A completed SPIRIT checklist is provided as Appendix X Relevant SPIRIT items are addressed throughout the Methods section below.

### Study design

This protocol describes a 2-arm, parallel-group randomized controlled trial with embedded implementation, qualitative, and health economic components. Families will be randomized to immediate S-FAME or to a comparator pathway consisting of treatment as usual during the study period and later access to P-FAME (Parental- Family-Centered Treatment Program for Problematic Gaming and Excessive Screen Use in a Clinical Child and Youth Population), a brief online parent intervention. Six social services units across participating municipalities have endorsed the project and will support implementation procedures.

### Recruitment and eligibility

Families will be recruited through participating municipal social services when parents or children seek support for repeated digital media use conflict, impaired routines, parental concern, or child functioning problems. The target group is families who experience digital media-related difficulties severe enough to seek structured support from social services. Based on power analyses appx 176 participants will be required to detect a small effect size (d = 0.03), accounting for potential attrition. Eligible families must include a child aged 10–14 years; at least one parent or legal guardian willing to participate (note: both child and parent participation is required for S-FAME delivery); reported concern related to gaming, social media use, other digital media activities, or screen-related family conflict; and sufficient Swedish language ability to participate in sessions and complete measures. Families will be excluded when acute safeguarding concerns, severe psychiatric instability, ongoing crisis, or another service need makes participation in a group-based preventive intervention inappropriate.

### Randomization and control group

After consent, eligibility confirmation, and baseline assessment, families will be randomized in a 1:1 ratio to immediate S-FAME or the comparator pathway. Allocation will be concealed from the assigning researcher until after eligibility confirmation and baseline assessment, using a centralized randomization procedure (e.g., sealed envelopes or web-based allocation). The control group will receive regular support from social services (treatment as usual) during the study period, reflecting ordinary municipal routines available to families. After the study period, families in the control group will be offered P-FAME, a three-session online intervention for parents. This delayed parent-focused offer is included for ethical and practical reasons and should be described consistently as part of the control condition rather than as the same intervention as S-FAME.

### Intervention: S-FAME

S-FAME is an adaptation of the FAME model for a municipal social services context [18]. To avoid conceptual confusion with the earlier clinical feasibility pilot, the intervention should be understood as an adapted implementation package rather than only as a list of therapeutic components. The shared core is family-centered work with digital media-related routines, conflict reduction, and parent-child communication. The social services adaptation concerns how families enter the program, who delivers it, how risk and safeguarding issues are handled, how the program is presented as early support, and how the model fits ordinary municipal resources.

The program is delivered by trained social services staff or other qualified municipal personnel using a manual, facilitator training, session materials, and fidelity-support procedures. Content may include psychoeducation about digital media and health, structured family discussions about expectations and boundaries, exercises that help families identify conflict triggers, strategies for emotion regulation and de-escalation, and routines that protect sleep, school attendance, and offline activities. The emphasis in this protocol is not that these components are novel in themselves, but that they are packaged, delivered, and evaluated within a new preventive service setting.

For a detailed description of the program theory and logic model, please see Werner, Kapetanovic, and Claesdotter-Knutsson [18].

### Outcomes and measures

Assessments will be completed at baseline before randomization, immediately after the intervention period, and 1 month after program completion.

Child-reported measures will include the Gaming and Social Media Questionnaire (GSMQ-9) [22], Kessler Psychological Distress Scale-6 (K6) [23], Parenting Children and Adolescents (PARCA) items [24], Family Check-Up (FCU) warmth and conflict items [25], the Insomnia Severity Index for Adolescents (ISI-A) [26] or the version specified by the research team, and background information including age, gender, height, weight, living situation, and prior or current [EC5] child mental health service contact.

Parent-reported measures will include parent version of GSMQ-9, PARCA, FCU warmth and conflict items, and any additional service use, cost, work absence, or family resource measures required for the health economic evaluation.

### Qualitative and implementation evaluation

The qualitative component will examine more than satisfaction with the program. It will investigate how families understand the offer of support from social services, whether S-FAME feels accessible and non-stigmatizing, how children and parents experience joint participation, and which parts of the program are most useful or difficult to apply at home. Approximately 15 to 20 children and 15 to 20 parents will be interviewed by trained research assistants [27] within one month after participation, with final sample size guided by information power and thematic saturation.

Focus groups with social workers and group leaders will examine implementation fit, training needs, fidelity, perceived appropriateness for the target group, burden on ordinary services, safeguarding boundaries, and the feasibility of offering S-FAME without a prior individual needs assessment when legally and locally appropriate. Notably, it is necessary to understand whether the model can become part of routine municipal preventive work.

### Health economic evaluation

The health economic evaluation will estimate the costs of preparing, training, delivering, supervising, and maintaining S-FAME in municipal services. If the outcome data support it, the analysis will compare costs with effects on prespecified child and family outcomes and will explore cost-effectiveness or cost-benefit from municipal and broader societal perspectives. Relevant cost categories may include facilitator time, training, materials, coordination, service use, parent work absence, and downstream support needs.

Health economic evaluations of preventive family interventions in social services typically adopt a societal perspective that extends beyond direct service costs to include productivity losses, downstream service use, and family burden [28]. In the present study, costs will be estimated using a micro-costing approach [29], capturing facilitator time, training and supervision, materials, and administrative coordination through structured cost logs and staff time records. If the primary effectiveness outcomes show a statistically or clinically meaningful between-group difference, an incremental cost-effectiveness analysis will be conducted, expressing results as cost per unit improvement on the primary child outcome measure. A cost-benefit analysis may additionally be explored if downstream service use data permit monetization of outcomes [30]. Uncertainty in cost and effect estimates will be addressed through probabilistic sensitivity analyses and presented using cost-effectiveness acceptability curves [30]. All cost data will be reported in Swedish kronor (SEK) and converted to Euro for international comparability, using the exchange rate at the time of analysis.

### Statistical analysis

Analyses will be conducted using SPSS, Stata, R, Mplus, AMOS, or equivalent software as appropriate for the final model specification. Descriptive analyses will summarize recruitment, baseline characteristics, retention, attendance, missingness, and adverse events. The primary effectiveness analysis should follow the intention-to-treat principle. Between-group change over time will be estimated using regression-based models or mixed-effects models that account for repeated measurements and baseline values. If families are nested within groups, facilitators, or municipalities, clustering will be considered in the model structure or sensitivity analyses.

Mediation or mechanism analyses may test whether changes in parenting practices, boundary setting, warmth, or conflict explain changes in child digital media-related outcomes. These analyses should be presented as exploratory unless the sample size and temporal ordering are sufficient for confirmatory causal mediation. Missing data will be examined, and multiple imputation or full-information maximum likelihood will be considered when assumptions are plausible. Qualitative data will be analyzed using reflexive thematic analysis [31] with procedures to support credibility, such as team coding, analytic memos, and discussion of discrepant cases.

### Ethical considerations

SPIRIT items 24–26 – Ethics and consent: Ethical approval, consent procedures, and participant confidentiality are described in this section. SPIRIT item 29 – Declaration of interests: See Conflicts of Interest. SPIRIT item 31 – Protocol amendments: Any future amendments will be submitted to the Swedish Ethical Review Authority and registered on ClinicalTrials.gov (NCT07245862). See Appendix X for the complete SPIRIT checklist.

Participation is expected to involve low risk but may include discussion of family conflict and sensitive routines. Written and verbal information will be provided to children, parents, and participating professionals. Consent and assent procedures will follow Swedish ethical requirements, including guardian consent for children younger than 15 years and attention to the child’s age and maturity. Participation will be voluntary, and families may withdraw without consequences for ordinary social services support.

Data will be pseudonymized or deidentified and stored securely according to the General Data Protection Regulation and the ethical approval. Ethical approval has been granted by the Swedish Ethical Review Authority (Dnr 2025-05262-01; September 29, 2025).

## Results

Recruitment is planned to begin in February 2026 and end in December 2026. Final quantitative, qualitative, implementation, and health economic analyses are planned for the first quarter of 2027 after completion of follow-up assessments, data validation, and qualitative coding.

## Discussion

### Principal expected contribution

The principal contribution of this trial is the evaluation of whether a family-centered intervention targeting multiple members of the family network — specifically children and parents — reduces screen-related child problems, family conflicts, and improves overall family functioning [7, 8]. In addition, the trial is expected to clarify to what extent improvements in child screen-related functioning occur alongside changes in parenting practices and parent-child relationships. Moreover, this trial will also generate implementation knowledge about staff training, recruitment, family engagement, fidelity, and adaptation across municipalities.

The trial is expected to clarify whether improvements in child screen-related functioning occur alongside changes in parenting practices and parent-child relationships. It will also generate implementation knowledge about staff training, recruitment procedures, family engagement, fidelity, and adaptation across municipalities. These elements are essential for establishing whether a family-centered model can be delivered with fidelity and at scale within ordinary municipal social services constraints.

### Limitations

Potential limitations include recruitment, reliance on self-report measures, short follow-up, variation in municipal implementation, and possible differences between families who seek support and those who do not. The comparator pathway may also vary across municipalities if treatment as usual is not standardized. These limitations should be addressed by transparent reporting of local procedures, monitoring of implementation fidelity and adaptations, and sensitivity analyses when feasible.

## Conclusions

S-FAME represents a family-centered, low-threshold intervention designed for delivery within Swedish municipal social services. By evaluating its effectiveness, implementation feasibility, and health economic consequences in a randomized controlled design, this study addresses a critical gap in the evidence base for preventive support for children and families experiencing digital media-related difficulties. The findings will inform how municipalities can provide early, accessible, and non-stigmatizing family support before problems escalate to require specialist psychiatric care. If effective and feasible, S-FAME has the potential to be scaled across Swedish municipalities and to contribute to the broader evidence base for preventive family-centered interventions in social services.

## Supplementary Information

Supplementary Material 1.

## Data Availability

No datasets were generated or analysed during the current study.

## Abbreviations

CAP: Child and Adolescent Psychiatry
CBA: Cost-Benefit Analysis
CEA: Cost-Effectiveness Analysis
FCU: Family Check-Up
GSMQ-9: Gaming and Social Media Questionnaire
HBSC: Health Behaviour in School-aged Children
ICD-11: International Classification of Diseases, 11th Revision
IGD: Internet Gaming Disorder
ISI-A: Insomnia Severity Index for Adolescents
PARCA: Parenting Children and Adolescents Scale
P-FAME: Parent-focused Digital FAME Intervention
RCT: Randomized Controlled Trial
SEM: Structural Equation Modeling
S-FAME: Social Services Family-Centered Program for Digital Media–Related Family Difficulties
SPIRIT: Standard Protocol Items: Recommendations for Interventional Trials
WHO: World Health Organization
