# Supplementary material for: S-FAME: a low-threshold municipal social services intervention for digital media–related family difficulties in children aged 10–14 years: protocol for a randomized controlled trial
Source: BMC Psychol. 2026 Aug 12;14:1181. doi: 10.1186/s40359-026-05302-x (PMC13463398; doi:10.1186/s40359-026-05302-x)
Supplement: Supplementary file 1 [file 40359_2026_5302_MOESM1_ESM.docx]

# SPIRIT 2013 Checklist: Recommended items to address in a clinical trial protocol and related documents

Completed for: S-FAME: A Low-Threshold Municipal Social Services Intervention for Digital Media–Related Family Difficulties in Children Aged 10–14 Years: Protocol for a Randomized Controlled Trial

| **Section/item** | **Item No** | **Description** | **Addressed on page number / section** |
| --- | --- | --- | --- |
| **Administrative information** | | | |
| Title | 1 | Descriptive title identifying the study design, population, interventions, and, if applicable, trial acronym | Title Page, page 1 |
| Trial registration | 2a | Trial identifier and registry name. If not yet registered, name of intended registry | Title Page, page 1; Abstract, page 2 |
| Trial registration | 2b | All items from the World Health Organization Trial Registration Data Set | Abstract / Trial Registration, page 2 |
| Protocol version | 3 | Date and version identifier | Title Page, page 1 |
| Funding | 4 | Sources and types of financial, material, and other support | Declarations / Funding, end of document |
| Roles and responsibilities | 5a | Names, affiliations, and roles of protocol contributors | Title Page, page 1; Declarations / Authors contributions |
| Roles and responsibilities | 5b | Name and contact information for the trial sponsor | Title Page, page 1 |
| Roles and responsibilities | 5c | Role of study sponsor and funders, if any, in study design; collection, management, analysis, and interpretation of data; writing of the report; and the decision to submit the report for publication | Declarations / Funding |
| Roles and responsibilities | 5d | Composition, roles, and responsibilities of the coordinating centre, steering committee, endpoint adjudication committee, data management team, and other individuals or groups overseeing the trial | Methods / Study Design, page 5 |
| **Introduction** | | | |
| Background and rationale | 6a | Description of research question and justification for undertaking the trial, including summary of relevant studies (published and unpublished) examining benefits and harms for each intervention | Introduction, pages 3-4 |
| Background and rationale | 6b | Explanation for choice of comparators | Methods / Randomization and Control Group, page 6 |
| Objectives | 7 | Specific objectives or hypotheses | Objectives, pages 4-5 |
| Trial design | 8 | Description of trial design including type of trial (eg, parallel group, crossover, factorial, single group), allocation ratio, and framework (eg, superiority, equivalence, noninferiority, exploratory) | Methods / Study Design, page 5 |
| **Methods: Participants, interventions, and outcomes** | | | |
| Study setting | 9 | Description of study settings (eg, community clinic, academic hospital) and list of countries where data will be collected. Reference to where list of study sites can be obtained | Methods / Study Design, page 5 |
| Eligibility criteria | 10 | Inclusion and exclusion criteria for participants. If applicable, eligibility criteria for study centres and individuals who will perform the interventions | Methods / Eligibility, pages 5-6 |
| Interventions | 11a | Interventions for each group with sufficient detail to allow replication, including how and when they will be administered | Methods / Intervention: S-FAME, page 6; Randomization and Control Group, page 6 |
| Interventions | 11b | Criteria for discontinuing or modifying allocated interventions for a given trial participant | Methods / Ethical Considerations, page 9 |
| Interventions | 11c | Strategies to improve adherence to intervention protocols, and any procedures for monitoring adherence | Methods / Intervention: S-FAME, page 6; Qualitative and Implementation Evaluation, page 8 |
| Interventions | 11d | Relevant concomitant care and interventions that are permitted or prohibited during the trial | Methods / Randomization and Control Group, page 6 |
| Outcomes | 12 | Primary, secondary, and other outcomes, including the specific measurement variable, analysis metric, method of aggregation, and time point for each outcome | Methods / Outcomes and Measures, pages 6-7 |
| Participant timeline | 13 | Time schedule of enrolment, interventions, and assessments | Methods / Outcomes and Measures, page 6; Discussion, page 9 |
| Sample size | 14 | Estimated number of participants needed to achieve study objectives and how it was determined | Methods / Sample Size, page 8 |
| Recruitment | 15 | Strategies for achieving adequate participant enrolment to reach target sample size | Methods / Recruitment, page 5 |
| **Methods: Assignment of interventions** | | | |
| Allocation: Sequence generation | 16a | Method of generating the allocation sequence (eg, computer-generated random numbers) | Methods / Randomization and Control Group, page 6 |
| Allocation: Allocation concealment mechanism | 16b | Mechanism of implementing the allocation sequence, describing any steps to conceal the sequence until interventions are assigned | Methods / Randomization and Control Group, page 6 |
| Allocation: Implementation | 16c | Who will generate the allocation sequence, who will enrol participants, and who will assign participants to interventions | Methods / Randomization and Control Group, page 6 |
| Blinding (masking) | 17a | Who will be blinded after assignment to interventions (eg, trial participants, care providers, outcome assessors, data analysts), and how | Methods / Outcomes and Measures, page 7 |
| Blinding (masking) | 17b | If blinded, circumstances under which unblinding is permissible, and procedure for revealing a participant's allocated intervention during the trial | N/A (Open-label psychosocial intervention) |
| **Methods: Data collection, management, and analysis** | | | |
| Data collection methods | 18a | Plans for assessment and collection of outcome, baseline, and other trial data, including any related processes to promote data quality | Methods / Outcomes and Measures, pages 6-7 |
| Data collection methods | 18b | Plans to promote participant retention and complete follow-up, including list of any outcome data to be collected for participants who discontinue or deviate from intervention protocols | Methods / Outcomes and Measures, pages 6-7; Statistical Analysis, page 8 |
| Data management | 19 | Plans for data entry, coding, security, and storage | Methods / Ethical Considerations, page 9 |
| Statistical methods | 20a | Statistical methods for analysing primary and secondary outcomes | Methods / Statistical Analysis, page 8 |
| Statistical methods | 20b | Methods for any additional analyses (eg, subgroup and adjusted analyses) | Methods / Statistical Analysis (Mediation), page 8 |
| Statistical methods | 20c | Definition of analysis population relating to protocol non-adherence (eg, as randomised analysis), and any statistical methods to handle missing data | Methods / Statistical Analysis (Intention-to-treat, missing data), page 8 |
| **Methods: Monitoring** | | | |
| Data monitoring | 21a | Composition of data monitoring committee (DMC); summary of its role and reporting structure | N/A (Low risk behavioural intervention) |
| Data monitoring | 21b | Description of any interim analyses and stopping guidelines | N/A |
| Harms | 22 | Plans for collecting, assessing, reporting, and managing solicited and spontaneously reported adverse events and other unintended effects of trial interventions | Methods / Ethical Considerations, page 9 |
| Auditing | 23 | Frequency and procedures for auditing trial conduct, if any | Methods / Qualitative and Implementation Evaluation, page 8 |
| **Ethics and dissemination** | | | |
| Research ethics approval | 24 | Plans for seeking research ethics committee/institutional review board (REC/IRB) approval | Title Page, page 1; Methods / Ethical Considerations, page 9 |
| Protocol amendments | 25 | Plans for communicating important protocol modifications to relevant parties | Methods / Ethical Considerations, page 9 |
| Consent or assent | 26a | Who will obtain informed consent or assent from potential trial participants or authorised surrogates, and how | Methods / Ethical Considerations, page 9 |
| Consent or assent | 26b | Additional consent provisions for collection and use of participant data and biological specimens in ancillary studies | N/A |
| Confidentiality | 27 | How personal information about potential and enrolled participants will be collected, shared, and maintained in order to protect confidentiality | Methods / Ethical Considerations, page 9 |
| Declaration of interests | 28 | Financial and other competing interests for principal investigators for the overall trial and each study site | Declarations / Competing interests, end of document |
| Access to data | 29 | Statement of who will have access to the final trial dataset, and disclosure of contractual agreements that limit such access for investigators | Declarations / Availability of data and materials |
| Ancillary and post-trial care | 30 | Provisions, if any, for ancillary and post-trial care, and for compensation to those who suffer harm from trial participation | Methods / Ethical Considerations (safeguarding), page 9 |
| Dissemination policy | 31a | Plans for investigators and sponsor to communicate trial results to participants, healthcare professionals, the public, and other relevant groups | Discussion / Principal Expected Contribution, page 10 |
| Dissemination policy | 31b | Authorship eligibility guidelines and any intended use of professional writers | Declarations / Authors contributions |
| Dissemination policy | 31c | Plans, if any, for granting public access to the full protocol, participant-level dataset, and statistical code | Declarations / Availability of data and materials |
| **Appendices** | | | |
| Informed consent materials | 32 | Model consent form and other related documentation given to participants and authorised surrogates | To be provided to Ethics Committee / as supplementary material |
| Biological specimens | 33 | Plans for collection, laboratory evaluation, and storage of biological specimens for genetic or molecular analysis | N/A |
